# The Primary Prevention of Atopy: Does Early Exposure to Cats and Dogs Prevent the Development of Allergy and Asthma in Children? A Comprehensive Analysis of the Literature

**DOI:** 10.3390/life13091859

**Published:** 2023-09-02

**Authors:** Cristiana Indolfi, Elisabetta D’Addio, Chiara Lucia Bencivenga, Giulio Rivetti, Irene Bettini, Amelia Licari, Sara Manti, Francesca Mori, Michele Miraglia del Giudice, Angela Klain

**Affiliations:** 1 Department of Woman, Child and General and Specialized Surgery, University of Campania Luigi Vanvitelli, 80138 Naples, Italy; cristianaind@hotmail.com (C.I.); elisabettadaddio3@gmail.com (E.D.); chiara.bencivenga03@gmail.com (C.L.B.); giuliorivetti94@gmail.com (G.R.); michele.miragliadelgiudice@unicampania.it (M.M.d.G.); 2 Specialty School of Pediatrics, Alma Mater Studiorum, University of Bologna, 40138 Bologna, Italy; irene.bettini@gmail.com; 3 Department of Clinical, Surgical, Diagnostic, and Pediatric Sciences, University of Pavia, 27100 Pavia, Italy; amelia.licari@unipv.it; 4 Pediatric Clinic, Fondazione IRCCS Policlinico San Matteo, 27100 Pavia, Italy; 5 Pediatric Unit, Department of Human and Pediatric Pathology “Gaetano Barresi”, AOUP G. Martino, University of Messina, Via Consolare Valeria, 1, 98124 Messina, Italy; saramanti@hotmail.it; 6 Allergy Unit, Meyer Children’s Hospital, IRCCS, 50139 Florence, Italy; framori76@gmail.com

**Keywords:** prevention, atopy, early exposure, pets, children, cat, dog, asthma, allergy

## Abstract

The current literature shows mixed results relating to the significance of environmental exposure, such as owning a pet, and the development of atopy in children. Our review aimed to collect the most recent evidence on the association between early-life cat and dog ownership and the development of allergy and asthma. A comprehensive literature review was performed using PubMed and Scopus. The search included the main keywords of our PICO: (((early exposure) AND (children) AND (allergy)) OR (asthma)) AND (dog) OR (cat). Randomized controlled trials (RCTs), case–control studies, clinical trials, meta-analyses, and systematic reviews of children within the last five years (2018–2023) were searched and included. During the search process, 52 articles from PubMed and 43 from Scopus were found. A total of 17 articles were deemed to be suitable and included. Fairly consistent results regarding early exposure to pets, in particular dogs, and the prevention of food allergies have been described. Furthermore, there seems to be a protective effect against allergy and asthma in relation to the number of pets owned. The likelihood of a child developing allergy and asthma seems to be influenced by various factors, including the child’s genetic background and early exposure to different environmental factors, including allergens that may interact with the gut microbiota and immune system.

## 1. Introduction

Researchers have long believed that early-life exposure to allergens increases the likelihood of developing allergies and asthma later in life [1,2]. According to the literature, the development of allergy and asthma is thought to be significantly influenced by early environmental and lifestyle variables. Still, avoiding allergens, including pets, as a strategy for preventing atopic conditions has recently been questioned. The prevalence of pet housekeeping has increased worldwide [3]. The most common household pets are dogs and cats. Approximately 23% of people live with a cat and 33% have a dog, according to a survey online with 27,000 participants from 22 industrialized nations [4]. A total of 6% of South Koreans own cats, compared to 57% of Russians, and 7% of South Koreans own dogs, compared to 66% of Argentinians; 26% of Europeans have cats, while 25% have dogs. Cat and dog ownership is at 25% and 38% in the United States [5]. In Italy, the percentage of families that own a dog is about 27.1%, equal to 5.9 million, while those with cats is 18.3%, approximately 4 million [6]. These pets have allergens in their fur and saliva that are easily dispersed throughout the environment. Allergen exposure occurs mainly in households with pets, but can also happen at schools, nurseries, hospitals, and in public spaces [7,8,9]. It has been found that the allergen levels of dogs and cats vary widely, with higher levels occurring in carpeted and upholstered areas [10]. The majority of allergens may be divided into five main protein categories: latherins, secretoglobins, kallikreins, and lipocalins. Cat allergy symptoms are mostly caused by Fel d1, a secretoglobin [11]. Sebaceous, anal, and salivary glands are the principal producers of Fel d 1, which is mainly found in the epidermis and fur. In dogs, the major allergens are Can f 1 and Can f 2, which are lipocalins, and Can f 3, a serum albumin. They can be found in dogs’ saliva, urine, serum, and dander [12]. These allergens can be carried by small particles suspended in the air and adhere well to clothing and upholstery. The threshold levels of allergens associated with sensitization are greater than 1 μg/g for cats and greater than 2 μg/g for dogs. In comparison, the threshold levels associated with asthma symptoms in sensitized individuals are greater than 8 μg/g for cats and greater than 10 μg/g for dogs [13]. In the PreventADALL mother–child cohort, including more than 2500 Norwegian and Swedish 3-month infants, children as young as 3 and 6 months old, some participants already showed allergic sensitization to cats and dogs (0.3% and 1% and 0.7% and 0.8%, respectively) [14,15]. 

The current literature shows mixed results regarding the role of environmental exposure in the development of atopy and asthma in children. The early introduction of food allergens (e.g., peanuts) has been proven to be successful in preventing the development of food allergies [16,17,18,19,20]. In this review, we wonder if early exposure to dogs and cats can be a protective factor against allergies and asthma in preschool- and school-aged children.

## 2. Materials and Methods

### 2.1. Structured Clinical Question

The search was pursued with a search string developed on the PICO model (P, population/patient; I, intervention/indicator; C, comparator/control; and O, outcome). In particular, our PICO string was:

In children (P), is early exposure to dogs or cats (I) effective, compared to no early exposure (C), in reducing the development of allergy or asthma (O).

### 2.2. Literature Search

Despite being narrative, the sources were found using a systematic search procedure [21]. The search was conducted on the online databases MEDLINE (PubMed) and Scopus between 1 April 2023 and 4 July 2023. We included: (1) randomized controlled trials (RCTs), systematic reviews, meta-analyses, case–control studies, and clinical trials; (2) articles conducted on children and adolescents (birth -18 years); and (3) articles published within the last five years (2018–2023). The exclusion criteria were: reviews, case reports, case series, publications solely in non-English language, and studies with no free full-text availability.

The search included the main keywords of our PICO: (((early exposure) AND (children) AND (allergy)) OR (asthma)) AND (dog) OR (cat). One author (A.K.) conducted the study selection and this was further cross-checked by another author (C.I.) for accuracy. Disagreements were iteratively discussed until an agreement was reached. The initial screening of the studies was performed by examining the titles and abstracts to determine their eligibility. The inclusion criteria were applied, and studies that did not fit were excluded. The full-text publications of the remaining research were examined to decide which ones should be included. 

## 3. Results

**Figure 1:**
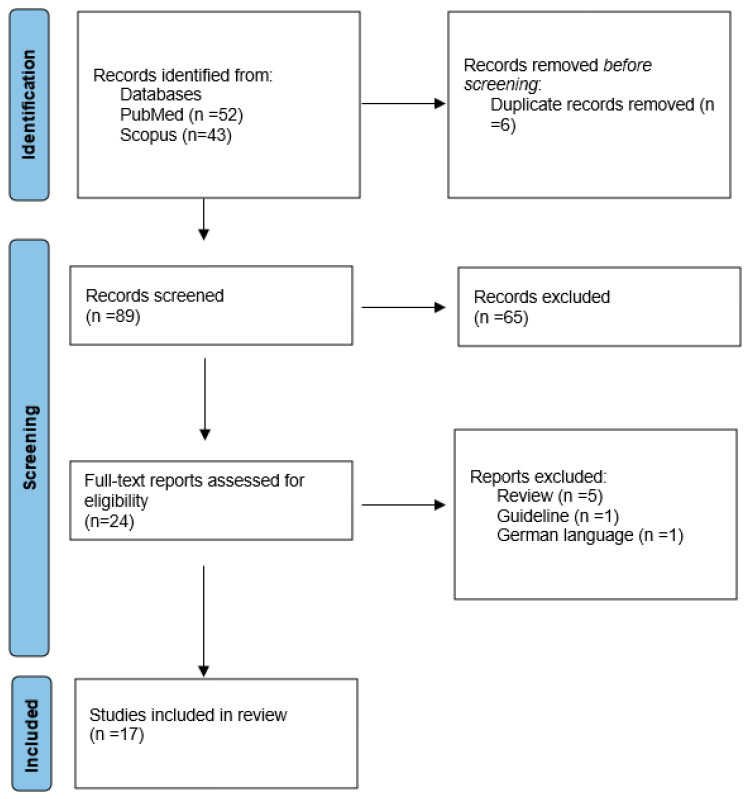
A diagram showing the selection process for articles.

The search string revealed 52 articles from PubMed and 43 articles from Scopus. A total of six duplicates were identified and removed. After excluding 72 articles (5 reviews, 1 guideline, 1 in German language, and 60 off-topic), 17 articles matched the inclusion requirements and were included in this review. The selection process is shown in Figure 1.

### Summary of the Included Study

Table 1 summarizes the studies included in this review [22,23,24,25,26,27,28,29,30,31,32,33,34,35,36,37,38]. [Table 1]. 

In recent years, the effectiveness of avoiding pets as a methodology for preventing atopy has come under scrutiny. We searched, in two of the significant scientific research platforms, articles regarding the association of early-life cat and dog ownership with allergy (food allergy, allergic rhinitis, and atopic dermatitis (AD)) and asthma in preschool and school-aged children, including the species (cat or dog), timeframe (prenatal and/or natal and/or infancy), role of allergic sensitization, and degree of ownership.

In our review, all the articles included explored the association between prenatal, natal, and/or infancy (1–2 years of life) exposure to cat and dog hair and the development of allergy and/or asthma in preschool and/or school-aged children [22,23,24,25,26,27,28,29,30,31,32,33,34,35,36,37,38]. Eight articles [27,28,29,30,31,32,33,36] investigated these outcomes in preschoolers. Three articles [24,25,33] explored the role of early pet households in the development of food allergy, supporting the hypothesis that early exposure to pets, particularly dogs, is protective against the development of food allergy. Six articles [22,23,27,30,31,32] investigating the role of atopic sensitizations show mixed results: three [23,27,31] showed that early-life dog and/or cat exposure may reduce the likelihood of developing allergic sensitization, but may contribute to the development of respiratory symptoms such as broncho-reactivity and non-atopic asthma [27]; two articles showed that early-life exposure to dogs [30] and cats [32] may intensify the risk of dog and cat sensitization, but dog sensitization may decrease the chance of AD [30]; and the metanalysis [22] found that owning cats or dogs did not show a significant association with allergic sensitization specific to cats or dogs. However, there was a strong association between allergic sensitization specific to cats or dogs and asthma in school-age children. With regard to the onset of asthma, two articles found no association between dog and cat housekeeping and the development of asthma in preschool and school-aged children [22,33]; three found that pet ownership was linked to the onset of nonatopic asthma and wheeze [27,28,36] in school- and preschool-age children; and three [31,34,37] found that keeping a dog/cat in the household in infancy was inversely associated with the chance of asthma (one article [34] found an association only for female dogs). Regarding the risk of developing AD, two studies discovered [24,33] no significant association; two reported that [28,36] pet keeping could increase the onset of eczema in preschoolers; and three manuscripts found [30,31,35] that pet ownership in infancy was inversely associated with a greater risk of AD in school-age children.

We found three articles [24,34,38] indicating an inverse dose–response relationship between keeping a pet in early infancy and the development of allergy and asthma, one [36] evidencing an increased risk, and one [22] reporting no association with asthma.

## 4. Discussion

### 4.1. Exposure to a Cat or Dog and the Protection against Allergies

In our work, we collected the most recent studies on the association between early-life cat and dog ownership and the development of allergy and asthma. We focused on early-life environmental exposure because strong evidence supports the existence of a time window during which environmental factors would be capable of shaping the immature immune system and gut microbiome [39,40,41]. Our analysis showed mixed results. However, a finding that emerged from the majority of the studies indicated a protective effect of early-life pet ownership, in particular dog ownership, on the onset of food allergy. Explanations can reside in the fact that having pets in the household during infancy leads to the modulation of the gut microbiome and a higher level of endotoxins, potentially offering protection against allergen sensitization through the enhancement of type 1 immunity [42,43]. However, the pathogenetic mechanisms through which such modulation would occur have not yet been completely clarified. In the study by Gern et al., the authors found that the presence of a dog during infancy increased IL-10 and IL-13 cytokine secretion patterns, as well as decreased allergic sensitization and AD [44]. These results propose that exposure to dogs after birth can impact immune maturation in a manner specific to genetic makeup, consequently lessening the likelihood of atopy development in susceptible children. 

An important role is also played by regulatory T (Treg) cells, which sustain allergen tolerance by governing both the innate and adaptive immune responses triggered by allergens [45]. The main mechanisms attributed to the establishment of tolerance in patients undergoing allergen-specific immunotherapy (AIT) involve the induction of Treg cells, coupled with a reduction in Th2 cell response and the elevation of allergen-specific IgG1, IgG4, and IgA levels [46]. New discoveries have indicated that residing in a farming setting and consuming farm milk, as well as exposure to numerous pets, factors linked to increased microbial diversity, have been observed to provide protection against allergy and asthma. This protective effect is notably achieved through the activation of Treg cells [47,48]. Maternal exposure to farm animals during fetal development is associated with an increased count of Treg cells in the umbilical cord blood and a decrease in the release of Th2 cytokines and lymphocyte proliferation upon innate stimulation [49]. Furthermore, research has demonstrated that the thymus-derived Treg (tTreg) levels in the venous blood during fetal development vary based on the levels of pet exposure and presence of atopic conditions [50]. 

In recent years, many efforts have been focused on delineating the role that epigenetics, the science that deals with gene–environment interactions, play in the development and progression of allergies. A recent study found a connection between childhood pet exposure and epigenetic changes, implying an epigenetic association between pet ownership and a reduced likelihood of developing allergic rhinitis in preschoolers [51].

### 4.2. The Role of Early Exposure to a Cat or Dog on the Development of Cat or Dog Allergy Later in Life

In our review, mixed results regarding early exposure to cats and dogs and the development of cat or dog allergy were found [22,23,27,30,31,32]. The avoidance of pet exposure as a strategy for preventing pet allergies has been questioned. Although pet ownership is considered to be the main risk factor for allergic sensitization, and, theoretically, no contact with a cat or dog ever means no allergy at all, cat and dog sensitization can occur also in subjects reporting any contact with these animals, as evidenced by Liccardi et al. [52]. In fact, pet exposure can also occur indirectly, as pet dander allergens can disseminate through aerodispersion, sedimentation, and passive transport. Thus, their presence is nearly ubiquitous [53,54]. The main explanation regarding the prevention of allergy in children exposed to cats and dogs in their first years of life is that early exposure to a sufficient quantity of allergens may train the immune system not to react allergically to those animals and become tolerant. The concept of ‘trained immunity’ was first coined in relation to infections or vaccinations. It refers to the functional, metabolic, and epigenetic adjustments that innate immune cells undergo due to prior stimuli, resulting in an ‘immunological memory’. As a result, innate immune cells exhibit an enhanced immune response upon subsequent stimulation, such as an increased release of cytokines [55]. Nowadays, this ‘trained immunity’ notion is applied to many disorders, including allergic diseases. Immune cell training can occur either systematically within the bone marrow or locally within tissues. In this scenario, various pathways become active and interconnected, with the release of many chemokines and cytokines (the cytokines thymic stromal lymphopoietin (TSLP), IL-25, IL-33, IL-4, IL-5, and IL-13)), orchestrating the initiation of trained immunity and, finally, resulting in the promotion of tolerance [56,57]. 

### 4.3. The Combination of Cat/Dog Exposure and Farming

The topic of how much our environment affects human health emerges in a world with fast-expanding urbanization and a loss of connection to nature. Respiratory illnesses, including allergies and asthma, have significantly increased in recent decades [58]. By 2050, the World Health Organisation (WHO) predicts that half of humanity will experience at least one allergic condition [59]. A causal relationship between people’s health and their environment appears likely in this situation. A protective environment model against atopy is supplied by farms that create the so-called ‘farm effect’ [60,61,62]. Rural life and frequently interacting with farm animals and other pets are protective against the development of allergies and asthma [63,64]. This theory corresponds to the so-called ‘hygiene hypothesis’: it has been suggested that extreme hygiene reduces the microbial stimulus necessary for the immune system to grow normally, causing an increased incidence of atopy [65]. Thus, children living in rural areas or with prolonged contact with animals are more likely to be infected and exposed to endotoxins in an unsanitary environment. Therefore, compared to others, they would experience fewer allergies by maintaining a healthy balanced immune system, where the T-helper 1 (Th1) response predominates on the Th2-driven proinflammatory state implicated in allergic reactions [66,67,68,69].

In a Polish study examining the impact of furry pets, such as cats and dogs, on the health of people with allergic diseases, 18617 individuals (16562 from urban and 2055 from rural areas) were included and asked to answer the European Community Respiratory Health Survey (ECRHS) and International Study of Asthma and Allergies in Childhood (ISAAC) study questionnaire. The results showed that keeping furry animals in rural regions prevented allergic disorders, while in cities, these animals increased the symptoms of bronchial asthma, the risk of cough (*p* = 0.02), and wheezing (*p* = 0.01) [70]. Therefore, it seems that other factors may predispose people to the development of allergies (i.e., delivery, antibiotic therapies, and the presence of siblings, etc.). 

**Figure 2:**
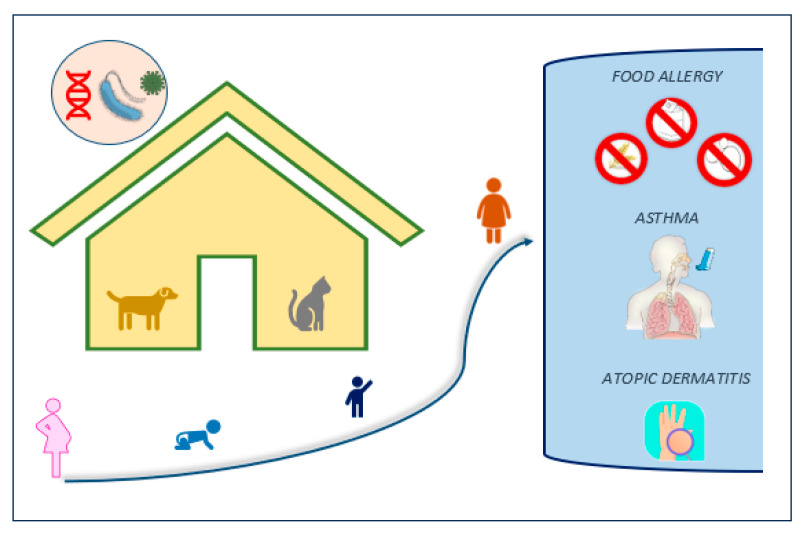
The set of environmental factors (i.e., early-life cat and dog ownership and rural life, etc.) and pathogens to which each individual is exposed during their life from the moment of conception (exposome), genetic factors, and the interaction between environment and genetics (epigenetics), represent a comprehensive model for explaining the development and the progression of diseases, including atopic conditions such as food allergy, allergic asthma, and atopic dermatitis.

In recent times, scientists have recommended the inclusion of the exposome concept—an all-encompassing spectrum of an individual’s environmental encounters, spanning from conception onwards. This encompasses allergens, external pollutants, tobacco smoke, the individual’s resident microbiota, lifestyle factors, rural/urban environment, climate change, and a broader spectrum of social, psychological, and economic influences. The exposome approach holds the potential to offer clearer insights into the interconnectedness of variables related to each other [71,72]. Precisely defining the external exposome on a molecular level, along with its potential significance as a clinically significant allergen, is crucial for understanding the diverse range of phenotypes within atopic conditions. This understanding can contribute to personalized diagnoses and treatment approaches [73]. Figure 2.

### 4.4. The Role of other Environmental and Genetic Factors on the Development of Allergy

The hygiene hypothesis cannot be the only explanation, considering the mixed results of the articles available in the literature and included in this review. Most of the results, including those in this review, do not provide strong evidence for a connection between owning a cat or dog and developing allergic sensitization to their specific allergens. This finding suggests that cat- or dog-related allergic sensitization is unlikely to be primarily caused by pet keeping. Instead, it is more likely that other factors, such as genetic susceptibility, may play a decisive role in this regard. Filaggrin (FLG) is essential for aligning keratin filaments, controlling keratinocyte shape, and maintaining epidermal texture. FLG genetic mutations represent a significant risk factor for AD and atopic march in children [74,75,76,77]. A recent article explored the link between allergy sensitization and FLG mutations in relation to allergen exposure [78]. The mite, cat, and dog allergen levels were assessed in a population-based birth cohort through dust samples obtained from their houses within the first year of life. Between birth and the age of 16, sensitization was evaluated six times. Children with FLG mutations were more likely to develop cat sensitization than those without them; moreover, a statistically significant (*p* = 0.035) interaction between FLG and Fel d 1 exposure was observed. Different interactions were identified for dogs: although there was a significant relationship between FLG and dog ownership, children with FLG mutations who were exposed to dogs as infants had a significantly lower risk of sensitization to any allergen (*p* = 0.03) [77]. These results underline that FLG mutations modify the association between allergen exposure and sensitization, with different effects at different ages and between other allergens. Moreover, sensitization to pets was associated with skin barrier dysfunction in children with AD [79]. 

Other mechanisms involved in the relationship between pet exposure and allergy concern the intestinal microbiota. Exposure to household furry pets may influence the gut microbiota of infants. A recent study revealed that prenatal dog exposure may increase the diversity of the gut bacteria in babies between the ages of three and six months old, especially formula-fed babies. Dog exposure was associated with Fusobacterium genera enrichment and the enrichment of *Collinsella, Ruminococcus, Clostridaceae,* and *Lachnospiraceae OTUs* [80]. A further study, conducted on 746 infants from the Canadian Healthy Infant Longitudinal Development Study (CHILD) cohort, showed that exposure to pets increased the abundance of two bacteria, *Ruminococcus* and *Oscillospira*, which have been negatively associated with childhood atopy [81]. Furthermore, the gut microbiota of infants differed following various birth scenarios: *Streptococcaceae* were significantly and substantially reduced by pet exposure among vaginally born infants exposed to intrapartum antibiotic prophylaxis (*p* 0.001), reflecting an 80% decreased likelihood of a high abundance (OR 0.20, 95%CI, 0.06–0.70) of pet exposure during pregnancy alone and a 69% reduced likelihood (OR 0.31, 95%CI, 0.16–0.58) for exposure in the pre-and postnatal periods [80].

Probably, the explanation of the association between dog- and cat-keeping before pregnancy and in the first years of life and allergy and asthma is much more complex than these results explain, because the concepts of allergy and asthma themselves encompass multiple phenotypes with diverse underlying aetiologies [82]. 

Furthermore, the duration and degree of pet ownership, the onset of ownership, and the level of exposure among non-pet-owning housekeepers should be subject to further investigation.

This review has some limitations that should be taken into account. One of the significant limitations is the PubMed and Scopus search approach, and the generalizability of the findings being limited by the heterogeneity of the study design, intervention characteristics, and methodology. 

However, our objective was to conduct a rapid synthesis of the recent evidence on the association between early exposure to cats and dogs and the development of allergy and asthma. PubMed and Scopus are standalone, reliable platforms for retrieving the most relevant publications effectively. Although the research methodology is systematic, our review is narrative. Moreover, as mentioned previously, allergic diseases and asthma include different phenotypes, with different trajectories, likely to have other environmental and genetic associations. 

## 5. Conclusions

Children encounter numerous environmental exposures during the early stages of life that can play vital roles in their overall development and well-being. In recent years, there has been growing skepticism regarding the effectiveness of pet avoidance as a strategy for preventing atopic diseases. In our review, despite the mixed results, we found reasonably consistent results regarding early exposure to pets, in particular dogs, and the prevention of food allergies. Further studies are needed to investigate the role of animal characteristics (such as sex, breed, and age), as well as environmental factors, such as geographic location, inner city and rural life, and genetic predisposing factors, in order to gain a deeper understanding of their influence on atopic diseases.

## Figures and Tables

**Table 1:** Articles examining the association between early exposure to cats and/or dogs and allergy and asthma in children.

Reference	Study Group	Study Type	Aim	Age of Outcome Assessment	Key Results
[22]	In the European Union Child Cohort Network, information was collected from 77,434 mother–child pairs from 9 birth cohorts.	Meta-analysis	To investigate whether early ownership of cats and dogs is associated with asthma in school-aged children.	The outcomes were evaluated in school-age children employing the International Study of Asthma and Allergy in Childhood (ISAAC) questionnaire.	No significant correlation was found between owning cats or dogs and the likelihood of developing asthma. The duration and the degree of ownership did not significantly affect these associations. Likewise, owning cats or dogs did not show a significant association with allergic sensitization specific to cats or dogs. However, there was a strong relation between allergic sensitization specific to cats or dogs and the presence of asthma in school-age children.
[23]	Data from 1231 children born in 1996–1997 at Östersund Hospital in Sweden were collected.	Observational study	To determine the relationship between dog and cat ownership during and after the first year of life and the likelihood of sensitization and allergy symptoms at age 13.	At 13 years of age, diagnoses of dog and cat allergies and asthma were made based on parental reports of allergic symptoms in a sample size of 834 individuals.	Keeping a dog or a cat during the first 12 months of life was found to decrease the risk of sensitization to dog or cat allergens, respectively, as well as sensitization to birch and other allergens (Timothy, Cat, Birch, Horse, Dog, Soy, Wheat, Fish, Egg, Milk). Furthermore, owning a cat during and after the first year of life decreased the likelihood of developing cat allergies and hay fever. Dog ownership beyond the first year of life had no effect on allergic symptoms, however, having a dog at home during the first 12 months of life lowered the likelihood of developing dog and cat allergies.
[24]	A randomized trial was conducted on 1303 infants aged three months to evaluate the prevention of food allergy. A survey was administered to determine pet ownership, and the patients were assessed for AD at the time of enrolment.	Randomized control study	To research how having a pet in the house might safeguard against food allergies.	Analyses of serum and skin were performed at 3 months, 1, and 3 years to evaluate sensitization to food and aeroallergens. Additionally, food allergy status was determined through double-blind, placebo-controlled food challenges conducted between 1 and 3 years of age.	Infants who lived with dogs exhibited a 90% lower likelihood of developing a food allergy. Notably, none of the 49 newborns who had at least two dogs in their household experienced food allergies, raising the possibility of a dose–response link. Specifically, for each additional dog owned, the odds of developing a food allergy decreased. However, no significant association was observed between owning dogs or cats and the development of AD.
[25]	Data from Japan Environment and Children’s Study, including 97,413 mother–child dyads, were evaluated.	Prospective study	To investigate the impact of pet exposure on the likelihood of developing food allergies.	The prevalence of food allergy in pre-schoolers was assessed based on questionnaires.	Exposure to dogs or cats during fetal development or the first years of life reduced the chance of developing food allergies up to the age of 3. Specifically, dog ownership was associated with a lower risk of developing egg, milk, and nut allergies, while cat ownership was linked with a lower risk of developing wheat and egg allergies.
[26]	Data from the Polish Mother and Child Cohort (REPRO_PL) were gathered.	Prospective study	The objective of the study was to investigate if early-life lifestyle variables and indoor allergen exposure affected a child’s chance of developing asthma, AD, and allergic rhinitis by the age of 10.	Data were collected at 10 years of age, using questionnaires during interviews.	Among the children included in the study, cat and dog allergens were the most commonly detected allergens in their homes, present in all of the households. A higher concentration of the cat allergen Fel d1 in house dust was found to be significantly associated with an increased risk of developing asthma at the age of 10 years. However, no significant associations were observed between allergen concentrations and the development of AD or allergic rhinitis. For AD and allergic rhinitis, no significant associations were observed.
[27]	The Cohort for Childhood Origin of Asthma and Allergic Diseases was used to provide information.	Prospective study	To examine the relationship between dog ownership at any point between pregnancy and the age of 1 year and sensitivity to airborne allergens at ages 3 and 7, bronchial hyperresponsiveness (BHR), and asthma at age 7.	At 36 weeks of gestation, at delivery, at 6 months, at 1 year, and then once a year, regular follow-up visits for medical examinations and self-report questionnaires were carried out. At 3 and 7 years old, BHR and asthma were evaluated, as well as sensitization to aeroallergens.	No significant differences in sensitization to dogs at ages 3 and 7 were observed between children who owned dogs and those who did not. However, owning a dog during early life was found to reduce the risk of sensitization to aeroallergens at age 7. On the other hand, dog ownership was associated with a significant increase in the risk of nonatopic BHR. Furthermore, dog ownership was linked to the development of nonatopic asthma at 7 years of age.
[28]	Parents of children aged 3 to 6 years (N = 3606) reported data on home environment and symptoms using ISAAC questionnaire in Taiyuan. The survey collected information on symptoms experienced during the first 2 years of life and symptoms reported within the past year.	Retrospective study	The study aimed to examine the reported appearance of childhood wheeze, rhinitis, and eczema symptoms concerning the prenatal, perinatal, and postnatal home environment.	Information on symptoms experienced during the first 2 years of life and symptoms reported within the past year were collected in preschool children.	The prevalence of wheezing and eczema was increased in households with dogs.
[29]	The study involved the participation of children aged 3 to 6 years from daycare centers in seven Chinese cities, with a total sample size of 39,782 children. Parents of children completed a questionnaire regarding the home environment and their children’s health, which included questions about rhinoconjunctivitis and wheezing in the presence of furry pets, as well as diagnosed rhinitis. Additionally, data from monitoring stations were used.	Retrospective study	To investigate how early-life exposure to hairy pets affects a child’s respiratory symptoms.	Outcomes were evaluated in preschool children aged 3 to 6 years.	Children who lived in rural or suburban regions and whose mothers worked as farmers during pregnancy had lower rates of other diagnosed rhinitis (unrelated to furry pets). Both perinatal and postnatal ownership of dogs and cats were associated with symptoms related to furry pets and diagnosed rhinitis specifically related to furry pets. In contrast, owning cats and dogs appeared to have a protective effect against other forms of diagnosed rhinitis.
[30]	Data from Shanghai Allergy Cohort were collected. Information on furry pet ownership was collected through questionnaires.	Prospective study	To research how having pets early in life affects children’s pet AD and sensitization.	Pet sensitization and AD were diagnosed at 5 years old.	Domestic pet ownership during infancy and preschool years was positively linked with a higher risk of dog sensitization. A greater risk of AD at age 5 was inversely related to pet ownership during infancy.
[31]	Information from 3781 children, including in the Finnish Type 1 Diabetes Prediction and Prevention (DIPP) Nutrition Study, was collected at age 5 using ISAAC questionnaire. Data on allergic disease and asthma and exposure to pets during the first 12 months of life were gathered.	Retrospective study	To examine whether exposure to farm and indoor pets in infancy affects the chance of developing asthma and allergies by age 5.	Asthma and allergy were assessed by age 5.	Asthma, allergic rhinitis, and atopic sensitization risk were found to be inversely correlated with the presence of dogs in the home. On the other hand, owning a cat was linked to a lower chance of developing atopic eczema.
[32]	From birth through to age 2 years, 108 mother–child couples included in the Kingston Allergy Birth Cohort were monitored.	Prospective study	To assess the impact of exposure to seven air pollution variables during pregnancy and the first 2 years of life on allergy sensitization.	A skin prick test (SPT) was performed on the 2-year-old children.	Exposure to cats during the 6-month period resulted in a significant increase in the OR of having a positive SPT result. No statistically significant associations between a positive SPT and dog exposure were found.
[33]	539 mother–child pairs included in the study were part of the Polish Mother and Child Cohort (REPRO_PL), a multicentre prospective cohort established in 2007. Mothers in each trimester of pregnancy and 1 year after childbirth completed a questionnaire on animal exposure.	Prospective study	To evaluate the association between prenatal and postnatal exposure to pet ownership and the development of AD, food allergy, and wheezing in children at the ages of 1 and 2.	Children’s health status was assessed at around one year and two years of age.	Keeping a dog at home before and during pregnancy decreased the risk of food allergy in the first year of life. On the other hand, keeping any animal other than a dog (cat, hamster, guinea pig, or rabbit) before and during pregnancy increased the risk of food allergy in the first year of life for children.
[34]	Records from 23585 Swedish children born from 2001 to 2004 were collected from national register.	Retrospective study	To evaluate if dog housekeeping during the first 12 months of life and different dog characteristics reduce the risk of asthma among school-age children.	The outcomes were evaluated at age 6.	Living with female dogs during the first year of life was associated with a lower risk of developing asthma compared to male dogs. Additionally, children who had 2 or more dogs had a decreased chance of asthma compared to those with only one dog.
[35]	Data from 756 children, aged 6–7, about exposure to dogs and cats and allergic diseases were collected with the ISAAC questionnaire.	Cross-sectional retrospective study	To analyze the influence of early exposure to dogs or cats on the prevalence of asthma and allergy among school-aged children.	Data regarding the prevalence of allergic diseases were collected at the age of 6–7 years old.	Exposure to dogs, whether indoors or outdoors, showed a significant association with a reduced prevalence of AD. On the other hand, exposure to outdoor cats was linked to nocturnal coughing and ongoing rhinitis.
[36]	Parental reports of 7360 children from birth to 8 years old in China were analyzed, using the ISAAC questionnaire.	Retrospective study	To find the relationship between pet keeping (cat, dog, rodent, bird, or fish) and asthma and allergy in infancy and childhood.	ISAAC questionnaire was used to assess the association between allergy and early-life pet exposure in children aged 0–8 years old.	Keeping pets, especially cats, was a notable risk factor for diagnosed asthma and AD. Exposure to a pet in early childhood notably heightened the risk of experiencing current wheeze, current dry cough, and diagnosed rhinitis. Even after accounting for avoidance behavior, the adverse impact of pet keeping on children’s health became even more evident. A dose–response relationship was observed between pet keeping and the prevalence of current wheeze and eczema.
[37]	Data from 442 children belonging to a birth cohort of high-risk inner-city children were collected to examine the effects of prenatal and early-life factors on the risk of asthma in school-age individuals.	Prospective study	To discover early-life environmental risk factors for asthma in children.	Data were analyzed at 7 years of age.	In the first 3 years of life, a decreased asthma risk was linked to higher levels of cockroach, mouse, and cat allergens in household dust.
[38]	Two Sweden cohorts were examined (n = 1029, n = 249). The first cohort consisted of 7- to 8-year-old children. The second cohort was a birth cohort. Data regarding asthma and allergy were collected through validated questionnaires in school-age children.	Cross-sectional and retrospective study	To determine if keeping cats and dogs throughout the first year of life was associated in a dose-dependent manner.	The onset of allergy and asthma disease was evaluated in school-age children (7–9 years old)	Between individuals without pets and those with five or more pets, the prevalence of allergy drastically dropped from 49 to 0%. Similarly, the prevalence of allergy in the last year decreased from 32% to zero with an increasing number of pets.

## Data Availability

Data sharing not applicable.
